# Clinical characteristics, management and health related quality of life in young to middle age adults with COVID-19

**DOI:** 10.1186/s12879-021-05841-1

**Published:** 2021-02-01

**Authors:** Chiara Temperoni, Stefania Grieco, Zeno Pasquini, Benedetta Canovari, Antonio Polenta, Umberto Gnudi, Roberto Montalti, Francesco Barchiesi

**Affiliations:** 1 Malattie Infettive, Azienda Ospedaliera Ospedali Riuniti Marche Nord, Pesaro, Italy; 2 Dipartimento di Scienze Biomediche e Sanità Pubblica, Università Politecnica delle Marche, Ancona, Italy; 3 Pronto Soccorso e Medicina d’Urgenza, Azienda Ospedaliera Ospedali Riuniti Marche Nord, Pesaro, Italy; 4 Dipartimento di Sanità Pubblica, Unità di Chirurgia Epato-bilio-pancreatica, Mininvasiva e Robotica, Università Federico II, Naples, Italy

**Keywords:** COVID-19, Young to middle age adults, SARS-Cov-2, Respiratory distress, Health related quality of life

## Abstract

**Background:**

The outbreak of COVID-19 has rapidly spread to Italy, including Pesaro-Urbino province. Data on young to middle age adults with COVID-19 are lacking. We report the characteristics, management and health-related quality of life (HRQoL) in patients with COVID-19 aging ≤50 years.

**Methods:**

A retrospective analysis was performed in all patients ≤50 years with a confirmed diagnosis of COVID-19 admitted to Emergency department (ED) of San Salvatore Hospital in Pesaro from February 28th to April 8th, 2020. Data were collected from electronical medical records. HRQoL was investigated after 1 month from hospital discharge using the SF-36 questionnaire. Outcomes were evaluated between hospitalized and not hospitalized patients.

**Results:**

Among 673 patients admitted to the ED and diagnosed with COVID-19, 104 (15%) were ≤ 50 years old: 74% were discharged at home within 48 h, 26% were hospitalized. Fever occurred in 90% of the cases followed by cough (56%) and dyspnoea (34%). The most frequent coexisting conditions were hypertension (11%), thyroid dysfunction (8%) and neurological and/or mental disorders [NMDs] (6%). Mean BMI was 27. Hypokalaemia and NMDs were significantly more common in patients who underwent mechanical ventilation. Regardless of hospitalization, there was an impairment in both the physical and mental functioning.

**Conclusions:**

Overweight and hypertension are frequent conditions in young to middle age adults with COVID-19. Hypokalaemia and NMDs are commonly associated with progressive disease. A significant impact on HRQoL in the early stage of post-discharge is common in this population.

## Background

In early December 2019, 41 cases of coronavirus disease 2019 (COVID-19) were described in Wuhan in Hubei Province [1]. The outbreak of the new pandemic coronavirus pneumonia has rapidly spread all over the world, included Europe and Italy, with an increasing number of cases. People have been facing this new virus changing their habits and their behaviours with a huge impact on mental and physical health [2].

**Fig. 1:**
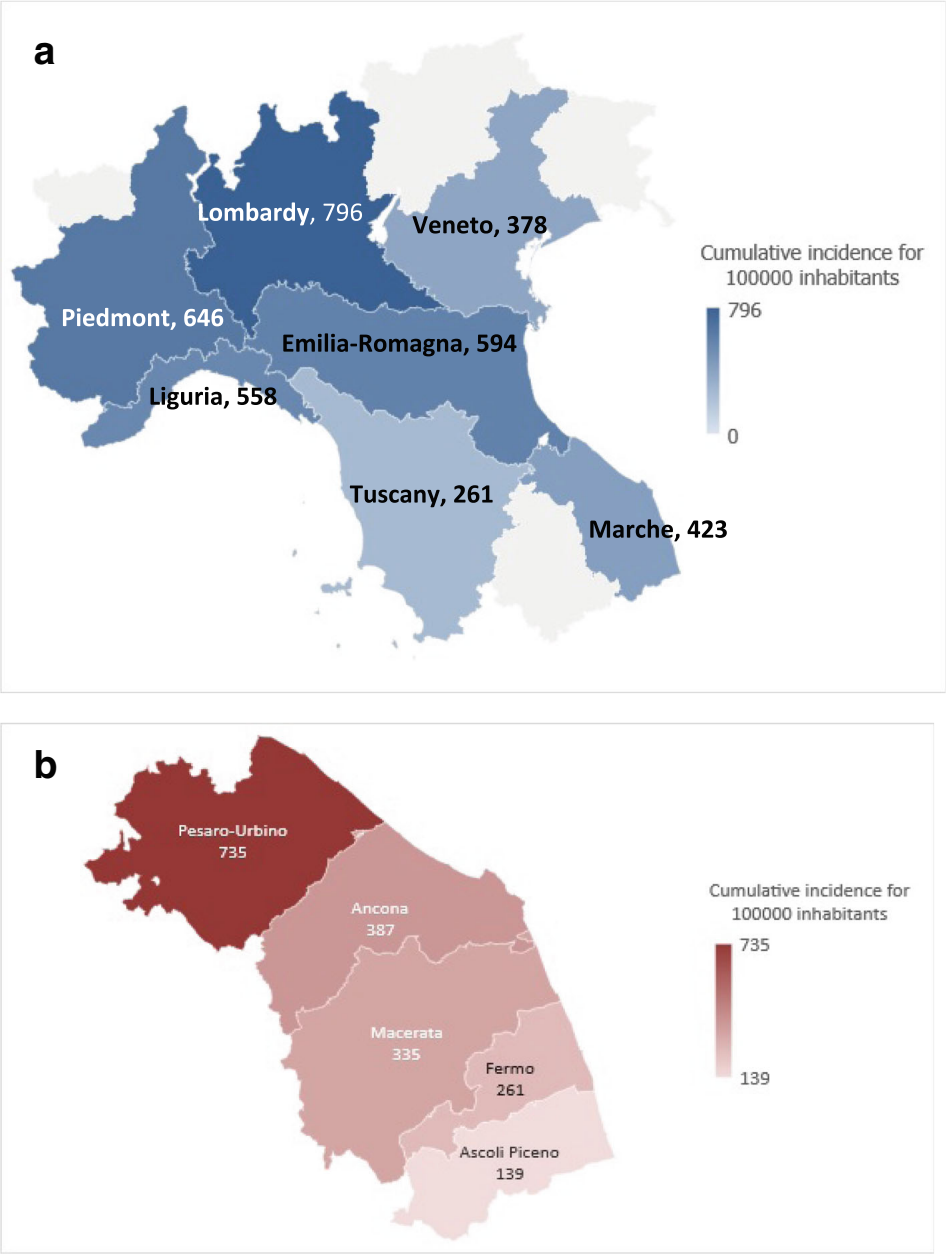
Distribution of Patients with Covid-19 from the seven italian regions mainly involved in the pandemic (**a**) and across Marche region (**b**). Laboratory-confirmed cases of Covid-19 throughout Northern and Center Italy according to the Italian Civil Protection as of May 8, 2020 (**a**) and across Marche region according to the GORES (Operative Regional Group for Sanitary Emergencies) as of May 8, 2020. Map produced by the Authors

The first two cases in Italy were reported on the 23rd of January 2020 coming from Wuhan [3]. Since then, severe acute respiratory syndrome coronavirus 2 (SARS-Cov-2) has overwhelmed Italy with approximately 215.000 infected subjects. Among the most affected areas in Italy (Fig. 1), Marche region counted almost 6.400 cases [4]. The median age of the Italian patients was 62 years old; patients between 19 and 50 years old represented the 28% of the infected population, while patients older than 50 were the 70% [4].

Data on adults younger than 50 years old with COVID-19 are lacking. Although one study from China reported a median age of 41 years, the overall population ranged from 41 to 65 years and it included even older patients [5]. Data from European countries describe patients who are generally older than those reported from Asiatic countries [6, 7]. Actually a remarkable interest of COVID-19 has been focused on older people who represent the main population at risk to develop SARS-Cov-2 related pneumonia [8–10]. Since little clinical information is available in patients with COVID-19 aging ≤50 years, the aim of this study was to illustrate the epidemiological, demographic, clinical, laboratory, radiological characteristics and clinical outcomes of laboratory-confirmed young to middle age patients with COVID-19.

All over the world measures, such as quarantine, containment and school and business closures, have been enforced to slow down virus spreading [11], this resulting in worsening of the quality of life, with an increasing of stress level and sedentary lifestyle [12] and lasting physical and psychological consequences [13]. Therefore, we also investigated the impact of COVID-19 in the quality of life of this population. To this aim we used the short form survey (SF-36) which includes 36 questions analysing eight health domains including physical functioning, role physical and bodily pain which evaluates physical sphere, mental health, role emotional, and social functioning items analysing mental component [14].

## Methods

### Patients

A retrospective analysis was performed on the confirmed cases of COVID-19, who were admitted to Emergency department (ED) of San Salvatore Hospital in Pesaro from February 28th to April 8th, 2020. All adults patients with age ranging from 18 to 50 years were considered in this study. A confirmed case of infection with SARS Cov-2 was defined by RT-PCR assay on nasopharyngeal swab.

### Data collection and definitions

Data were extracted from electronic medical records including patient demographic information, tobacco smoke addiction, underlying comorbidities, triage vital signs, referred symptoms on admission and the interval time lapse between illness onset and ED access. Fever was defined as axillary temperature of at least 37.5 °C. Respiratory distress syndrome was defined as PaO_2_/FiO_2_ ratio ≤ 300 according to the Berlin Definition [15]. Laboratory tests and radiological data on admission were also collected.

### Short form health survey (SF-36)

After 1 month from hospital discharge patients were interviewed and requested to answer to the short form health survey (SF-36). The SF-36 is an internationally instrument to measure Health-Related Quality of Life (HRQoL) [14]. It includes 36 questions analysing eight health domains including physical functioning, role physical and bodily pain which evaluates physical sphere, mental health, role emotional, and social functioning items analysing mental component. Scores for each domain can range from 0 (worst) to 100 (best), higher scores indicate better HRQoL. The SF- 36 has been used in many different diseases to evaluate the quality of life for patients with other respiratory infections such as Middle East Respiratory Syndrome (MERS) [16] and SARS-CoV-1 [17].

### Statistical analysis

Continuous variables were expressed as median (IQR) and compared with the Mann-Whitney U test or independent group *t* tests, when data were normally distributed; categorical variables were expressed as number (%) and compared by χ^2^ test or Fisher’s exact test. Comparison analysis was carried out between hospitalized and not hospitalized patients (i.e.: discharged at home within 48 h upon ED arrival). A two-sided α of less than 0.05 was considered statistically significant. All the statistical analyses were supported by SPSS (Statistical Package for the Social Sciences) version 25.0 software (SPSS Inc).

## Results

Among 673 patients admitted to the ED and diagnosed with COVID-19 from February 28th to April 8th, 2020, 104 (15%) were ≤ 50 years old. Demographic, clinical, laboratory and radiological characteristics of the patients are shown in Table 1. Age ranged from 22 to 50 years with a mean of 41 years, the majority were men and the mean of BMI was 27. Hypertension was the most frequent coexisting condition being observed in 11% of the patients, followed by thyroid dysfunction (8%), and neurological and/or mental disorders (6%). Mean days from illness onset to first hospital access was 8.8. Common symptoms at the onset were fever (90%), cough (56%) and dyspnoea (34%), less common symptoms were fatigue (17%), anosmia (16%), diarrhoea (15%) and chest pain (14%). Respiratory distress was present in 13% of the patients. Chest X-ray and/or CT scan revealed ground glass opacity, bilateral patch shadow or focal lesions in 27, 37 and 10% of the patients, respectively. In 26% of the cases, chest X-ray was negative.

**Table 1:** Demographic, clinical, laboratory and radiological characteristics of 104 young adults with COViD-19 considered in this study

Characteristics	All patients (*n* = 104)	Outpatients (*n* = 71)	Inpatients (*n* = 33)	*p* value
Mean age ± SD – years	41.1 ± 7.4	39.5 ± 7.5	44.8 ± 5.8	< 0.001
Male gender – no. (%)	56 (53.8%)	37 (52.1%)	19 (57.6%)	0.757
Healthcare worker – no. (%)	13 (12.5%)	11 (15.5%)	2 (6.1%)	0.218
Mean BMI (Body mass index) ± SD	27.1 ± 5.01	26.37 ± 5.12	28.6 ± 4.46	0.029
Smoking habit – no. (%)	11 (10.6%)	9 (13.8%)	2 (7.1%)	0.495
Coexisting conditions				
Hypertension	11 (10.6%)	8 (11.3%)	3 (9.1%)	> 0.999
Diabetes	4 (3.8%)	2 (2.8%)	2 (6.1%)	0.590
Chronic obstructive pulmonary disease	2 (1.9%)	2 (2.8%)	0	> 0.999
Cerebrovascular disease	1 (1%)	1 (1.4%)	0	> 0.999
Chronic liver disease	3 (2.9%)	2 (2.8%)	1 (3%)	> 0.999
Neurological disease and mental disorder	6 (5.8%)	2 (2.8%)	4 (12.1%)	0.079
Malignancy	3 (2.9%)	3 (4.2%)	0	0.550
Thyroid diseases	8 (7.7%)	7 (9.9%)	1 (3%)	0.431
Days from illness onset to visit hospital	8.8 ± 6.05	8.5 ± 6.49	8.5 ± 6.07	0.996
Signs and symptoms at the onset				
Fever	94 (90.4%)	61 (85.9%)	33 (100%)	0.028
Cough	58 (55.8%)	35 (49.3%)	23 (69.7%)	0.082
Dyspnoea	35 (33.7%)	18 (25.4%)	17 (51.5%)	0.016
Chest pain	15 (14.4%)	9 (12.7%)	6 (18.2%)	0.551
Fatigue	18 (17.3%)	13 (18.3%)	5 (15.2%)	0.906
Sore throat	9 (8.7%)	9 (12.7%)	0	0.054
Anosmia	17 (16.3%)	14 (19.7%)	3 (9.1%)	0.280
Diarrhoea	16 (15.4%)	9 (12.7%)	7 (21.2%)	0.406
Vomiting	5 (4.8%)	4 (5.6%)	1 (3%)	> 0.999
Headache	8 (7.7%)	6 (8.5%)	2 (6.1%)	> 0.999
Myalgia	11 (10.6%)	7 (9.9%)	4 (12.1%)	0.740
Syncope	6 (5.8%)	4 (5.6%)	2 (6.1%)	> 0.999
Respiratory distress syndrome	14 (13.95%)	2 (2.8%)	12 (36.4%)	< 0.001
Vital signs				
Systolic blood pressure. mm Hg	96 ± 15.51	128 ± 14	132 ± 18.1	0.289
Heart rate	91.7 ± 17.45	90.3 ± 18.3	94.6 ± 15.6	0.329
Respiratory rate	18 (17–24)	17 (16–18)	18 (17–24)	0.171
Laboratory findings				
White blood cell count, × 109/L (normal range 4–11)	5.820 ± 2.489	5.614 ± 2.259	6.264 ± 2.913	0.224
Lymphocyte count, ×109/L (normal range 1–4)	1.318 ± 0.609	1.415 ± 0.597	1.109 ± 0.592	0.018
Platelet count, ×109/L (normal range 150–400)	186 (152–248)	183 (161–246)	190.5 (142–250)	0.693
Alanine aminotransferase, U/L (normal range 0–35)	28 (18–40)	21 (15–33)	36.2 (26.7–50)	0.001
Aspartate aminotransferase, U/L (normal range 0–35)	24 (42–20.5)	24 (19–30)	42 (29.7–51)	< 0.001
Creatinine, mg/dl (normal range 0.67–1.17)	0.844 ± 0.21	0.823 ± 0.19	0.890 ± 0.24	0.128
Potassium, mEq/L (normal range 3.5–5.1)	3.99 ± 0.36	3.96 ± 0.348	4.05 ± 0.38	0.248
Lactate dehydrogenase, U/L (normal range 0–247)	250.5 (176.5–326.5)	179 (150–221)	317 (259–448)	< 0.001
Creatine kinase, U/L (normal range 0–195)	73 (49.25–124.5)	67.5 (49–90.7)	116 (59–270)	0.034
Procalcitonin ng/mL (normal range 0.38)	0.03 (0.02–0.08)	0.02 (0.02–0.04)	0.07 (0.02–0.132)	0.018
C-reactive protein mg/mL (normal range 0–0.49)	1.74 (0.49–5.72)	0.89 (0.195–2.44)	7.63 (2.66–11.72)	< 0.001
D-dimer, ng/L (normal range 0–500)	587 (298–920)	406 (263–494)	861 (591–1165)	0.001
Involvement on chest radiographs				
No involvement	27 (26%)	26 (37.7%)	1 (3%)	< 0.001
Ground-glass opacity	28 (26.9%)	23 (33.3%)	5 (15.2%)	
Bilateral lung patch shadow	37 (35.6%)	12 (17.4%)	25 (75.8%)	
Focal lesions	10 (9.6%)	8 (11.6%)	2 (6.1%)	

Data are expressed as mean ± SD, median (IQR) or n (%)

*p* values indicate differences between out and in-patients. *P* < .05 was considered statistically significant

In brackets are expressed percentages and IQR

Seventy-one patients (74%) were managed in ED and discharged at home within 48 h, 33 patients (26%) were hospitalized. Compared with patients who did not require hospitalization, in-patients were significantly older and were more likely to be overweight. Fever and dyspnoea were significantly more common in hospitalized patients. As expected, a significantly higher proportion of hospitalized patients had respiratory distress. Additionally, this group was more likely to have lymphocytopenia, hepatic disfunction, higher inflammation biomarkers (i.e.: PCT, CRP and D-dimer [p ranging from < 0.001 to 0.034]), and more extensive lung involvement (*p* < 0.001).

Six out of 33 hospitalized patients (18%) required mechanical ventilation (Table 2). Respiratory distress syndrome and hypokalaemia at the infection onset were significantly more common in patients requiring mechanical ventilation (p 0.001 and 0.028, respectively). No difference was noticed in other laboratory findings between patients who required and did not require ICU care. Among coexisting conditions, only neurological and/or mental disorders were significantly more common in patients requiring ICU care (*p* = 0.014). Table 3 details clinical features of six patients who required ICU care. Except for patient n. 5, who did not suffer from any underlying disease, the remaining five patients died from one to 39 days upon the admission in ICU.

**Table 2:** Demographic, clinical, laboratory and radiological characteristics of 33 hospitalized young adults with COViD-19 considered in this study

Characteristics	No ICU care (*n* = 27)	ICU care (*n* = 6)	*p* value
Mean age ± SD – years	45.48 ± 5.09	42.5 ± 8.26	0.257
Male gender – no. (%)	16 (59.3%)	3 (50%)	> 0.999
Healthcare worker – no. (%)	2 (7.4%	0	> 0.999
Mean BMI ± SD	28.7 ± 4.8	28.6 ± 2.84	0.989
Smoking habit – no. (%)	1 (4%)	1 (33.3%)	0.206
Coexisting conditions			
Hypertension	3 (11%)	0	> 0.999
Diabetes	2 (7.4%)	0	> 0.999
Chronic liver disease	1 (3.7%)	0	> 0.999
Neurological disease and mental disorder	1 (3.7%)	3 (50%)	0.014
Thyroid diseases	0	1 (16.7%)	0.182
Signs and symptoms at the onset			
Fever	100%	100%	–
Cough	21 (77.8%)	2 (33.3%)	0.053
Dyspnoea	13 (48.1%)	4 (66.7%)	0.656
Chest pain	6 (22.2%)	0	0.563
Fatigue	5 (18.5%)	0	0.556
Anosmia	3 (11.1%)	0	> 0.999
Diarrhoea	6 (22.2%)	1 (16.7%)	> 0.999
Vomiting	1 (3.7%)	0	> 0.999
Headache	1 (3.7%)	1 (16.7%)	0.335
Myalgia	4 (14.8%)	0	> 0.999
Syncope	6 (22.2%)	1 (16.7%)	0.335
Respiratory distress syndrome	6 (22.2%)	6 (100%)	0.001
Vital signs			
Systolic blood pressure. mm Hg	132 ± 18.1	131 ± 20	0.895
Heart rate	93.3 ± 16.3	103 ± 3.51	0.291
Laboratory findings			
White blood cell count, ×109/L (normal range 4–11)	6.437 ± 2.981	5.511 ± 2.714	0.492
Lymphocyte count, ×109/L (normal range 1–4)	1.091 ± 0.460	1.183 ± 1.051	0.739
Platelet count, × 109/L (normal range 150–400)	199(144.750–260.500)	156.5 (138.750–181.750)	0.308
Alanine aminotransferase, U/L (normal range 0–35)	39 (30.2–52)	25 (17.5–28.7)	0.055
Aspartate aminotransferase, U/L (normal range 0–35)	43 (28.5–49.7)	35 (30.7–67)	0.906
Creatinine, mg/dl (normal range 0.67–1.17)	0.86 ± 0.218	1 ± 0.294	0.093
Potassium, mEq/L (normal range 3.5–5.1)	4.1 ± 0.332	3.7 ± 0.460	0.028
Lactate dehydrogenase, U/L (normal range 0–247)	297 (255–365)	483 (362–729)	0.141
C-reactive protein mg/mL (normal range 0–0.49)	6.5 (2.55–11.25)	11.7 (11.1–14.1)	0.097
Involvement on chest radiographs			
No involvement	1 (3.7%)	0 (0%)	0.865
Ground-glass opacity	4 (14.8%)	1 (16.7%)	
Bilateral lung patch shadow	20 (74.1%)	5 (83.3%)	
Focal lesions	2 (7.4%)	0 (0%)	

Data are expressed as mean ± SD, median (IQR) or n (%)

*p* values indicate differences between out and in-patients. *P* < .05 was considered statistically significant

In brackets are expressed percentages and IQR

**Table 3:** Demographic, underlying diseases and outcome of six patients admitted to Intensive Care Unit

Case no.	Gender	Underlying disease	Home treatment	Days from hospital admission to ICU care	Length of stay in ICU (days)	Outcome
1	F	*Friedreich’s ataxia*	No treatment	–	1	Death
2	M	Epilepsy, intellectual disability, obesity	clonazepam, carbamazepine, olanzapine	6	19	Death
3	M	Duodenal ulcer, obesity	Esomeprazole	2	10	Death
4	M	Depressive disorder, obesity	Alprazolam	0	39	Death
5	F	No disease	No treatment	1	27	Discharged
6	F	Blindness, epilepsy, hypothyroidism	sodium valproate, clobazam, topiramate, levothyroxine	0	9	Death

Among 104 patients, 85 were contacted 1 month from hospital discharge and requested to answer to SF-36 questionnaire. A total of 64 subjects (75%) answered the SF-36 questionnaire. The results of the survey are reported in Table 4. HRQoL revealed that physical functioning, general health and mental health reached the highest scores (74, 63, and 59, respectively) while physical role, vitality, social functioning and emotional role reached the lowest scores (30, 48, 45 and 46, respectively). Additionally, there were no significant differences between hospitalized and not hospitalized patients in physical component or mental component scores.

**Table 4:** Average score of SF36* components reported by 64 COVID-19 patients

	All patients (*n* = 64)	Outpatients (*n* = 49)	Inpatients (*n* = 15)	*p* value
Physical functioning	74.3 ± 25.48	76.22 ± 25.34	68 ± 25.76	0.277
Physical role	30.47 ± 42.13	31.12 ± 42.86	28.33 ± 41.04	0.825
Bodily pain	54.34 ± 30.39	51.88 ± 28.50	62.40 ± 35.80	0.244
General health	63.06 ± 17.91	62.10 ± 19.37	66.27 ± 12.19	0.436
Vitality	48.44 ± 23.20	47.65 ± 21.22	51 ± 29.47	0.629
Social functioning	45.12 ± 29.52	43.62 ± 27.85	50 ± 35.04	0.468
Emotional role	46.87 ± 45.50	45.57 ± 45.99	51.11 ± 45.19	0.684
Mental health	59.06 ± 20.35	58.04 ± 20.38	62.40 ± 20.61	0.472
Physical component summary	49.87 ± 24.25	55.32 ± 23.48	56.25 ± 23.15	0.894
Mental component summary	55.54 ± 23.22	48.72 ± 23.14	53.63 ± 28.11	0.498

The SF-36 is an internationally instrument to measure Health-Related Quality of Life (HRQoL). It includes 36 questions analysing eight health domains including physical functioning, role physical and bodily pain which evaluates physical sphere, mental health, role emotional, and social functioning items analysing mental component. Scores for each domain can range from 0 (worst) to 100 (best), higher scores indicate better HRQoL

## Discussion

Overall, we showed that overweight and hypertension are frequent conditions in young to middle age adults with COVID-19, hypokalaemia and NMDs are instead commonly associated with progressive disease. A significant impact on HRQoL in the early stage of post-discharge is common in this population.

This study focused on clinical characteristics, management and health related quality of life in young to middle age adults with COVID-19 admitted to the ED of Pesaro Hospital. During the pandemic, Marche, and particularly the Province of Pesaro-Urbino, was one of the most affected regions in Italy.

Overall, our data highlight distinctive features of COVID-19 in this population.

First, as many as 26% of the patients was hospitalized upon arrival to the ED. This is a remarkable percentage considering the age. Even if there is a lack of data describing the management of patients after ED access, it is reasonable to think, looking at the regional prevalence of SARS-CoV-2, that many patients with mild symptoms were managed at home according to WHO indications [18]. Second, in contrast to many reports in which SARS CoV-2 seems to affect more males then females, our population included approximately an equal number of men and women. Conversely, we observed a slightly higher number of men (57%) requiring hospitalization after ED access. It has been demonstrated that for SARS-CoV-2, as for other similar infections (i.e.: MERS and SARS-CoV-1), the male gender is more affected than female thereby reflecting sex predisposition associated with genetic factors [19]. Third, several coexisting conditions were quite frequent in this population. In concert with other studies focused on patients with COVID-19 without age selection, an increase of BMI even in young to middle age adults has been observed (mean BMI SD 27.1 ± 5.01 kg/m^2^). As it has been already demonstrated in Influenza A virus [20], obesity may worsen the severity of respiratory diseases. One study showed that SARS-CoV-2 patients having BMI ≥35 are at higher risk of mechanical ventilation, compared to those with BMI < 25 [21]. This could be due to multiple factors. Accumulation of adipose tissue in the mediastinum and in the abdominal cavities seen in obese subjects determines lung mechanical dysfunction [22, 23]. Additionally, fat causes an abnormal cytokine production and an increasing inflammatory pathway activation thereby favouring the infection per se and worsening its clinical course.

Hypertension is one of the most frequent underlying diseases in patients with COVID-19 [24]. In our study, 11% of the patients suffered from this clinical condition. Although hypertension has been commonly described to increase the severity illness in patients with COVID-19 [25], it is still unclear whether hypertensive subjects are more likely to be infected by coronavirus. It is reasonable to think that angiotensin-converting enzyme 2 expression, frequently increased in these patients, and the activation of the renin-angiotensin system can be involved either in the entrance of the virus into the cell or in the inflammatory response [26]. Further studies are warranted to elucidate this issue.

Thyroid dysfunction was seen in 8% of our patients. Little is known about the correlation between COVID-19 and thyroid dysfunction. Thyroid hormones play an important role in regulating the immune response and in modulating pulmonary system and alveolar ventilation. Hypothyroid patients can have a decreased lung function [27] but there is no evidence that those who have a thyroid disorder, unless they are under immunosuppressive treatment, are at higher risk to be infected by coronavirus [28].

Fourth, we identified several features more frequently associated with young to middle age patients requiring ICU admission, namely the respiratory distress syndrome, the hypokalaemia and neurological diseases and mental disorders. While the more severe respiratory syndrome the greater risk of mechanical ventilation is easily explained, the relationship between the other two parameters and ICU admission is less clear.

Hypokalaemia has been already reported among patients with COVID-19 with progressive disease [29]. It can occur first through virus action on angiotensin-converting enzyme 2 with an increased potassium excretion by the kidneys and secondly through loss, with vomiting or diarrhoea, in patients with gastrointestinal symptoms [30]. Hypokalemia might worsen acute respiratory distress syndrome and acute cardiac injury, which are common complications in COVID-19 [29, 30].

It has already demonstrated that people with severe mental illness have a higher risk to develop pneumonia [31]. Lee et al., underline as patients with underlying mental health disease have higher risk for severe clinical outcomes of COVID-19 [32]. Poor information on the effect of chronic benzodiazepines use in patients with COVID-19 infection is available. It is interesting to note how four out of six patients, who underwent mechanical ventilation, were taking benzodiazepines. The mechanism of action of these drugs is enhancing the effect of γ-amino-butyric acid type A (GABA_A_) at the GABA_A_ receptors. Chronic benzodiazepine exposure could be associated with an increased risk of developing pneumonia [33] as GABA can play an important role in regulating the secretion of a great number of cytokines [34, 35].

A severe respiratory infection generally affects HRQoL. This has been demonstrated in subjects recovering from MERS [16], SARS-CoV-1 [17] and H1N1 [36]. Batawi et al. [16] demonstrated that subjects with MERS experiencing ICU admission scored low values for physical function, general health, vitality, emotional role and physical components. To our knowledge, there are only few reports considering the impact of COVID-19 on mental health and quality of life among these patients. Hu et al., evaluated the mental health status of 85 hospitalized patients (mean age 49 years) with COVID-19 [37]. They found that female sex, disease duration, levels of inflammatory markers and self-perceived illness severity were factors significantly related with mental disturbances. Liu et al., investigated the distress levels within young adults (18–30 years) during the COVID-19 pandemic [38]. They reported significant depression, anxiety and post-traumatic stress disorder during the first few weeks of the pandemic. In particular, a pre-existing mental health diagnosis makes this population more vulnerable to poorer quality of life. Despite a quite young age population analysed in this study and the majority of patients who were discharged early after ED arrival, we observed lowest rating scores in items regarding physical role, vitality, social functioning and emotional role. It is interesting to note how the quality of life reported by hospitalized patients did not differ from non-hospitalized ones, as shown by similar physical and mental component summary scores (around 50 in both groups). This can be due by the fact that patients discharged early from ED experienced the lockdown period, so their psychological and physical spheres were possibly affected as the ones hospitalized. One study [37] found a significant correlation between levels of inflammatory markers and physical and mental quality of life; although we also tried to investigate a possible correlation between laboratory markers, pre-existing pathologies and HRQoL scores, we did not find any significant relationship in our population (data no shown).

The present study has some limitations. First, being a single-centre study, the number of patients considered is low. The suspected but undiagnosed cases were ruled out in the analyses. This feature has certainly weakened the statistical power of the study. Nevertheless, we considered all patients admitted to the ED of Pesaro Hospital in a very limited time which represented the period with highest COVID-19 incidence in our country. Second, this was a retrospective analysis. Although we tried to collect as many clinical data as possible, we may have still missed useful information for the management of these patients. In particular, due to the massive burden of patients admitted at the ED, several laboratory parameters (i.e.: D-dimer, ferritin, IL-6 etc.) or second level radiological examinations (i.e.: CT scan) were not always performed, mainly at the beginning of the pandemic period. Third, we performed only one early SF-36 survey (within 1 month from hospital discharge), while late and repeated surveys (i.e.: three or six months thereafter) might be more useful either in differentiating HRQoL based on severity illness or showing a quality of life improvement. Finally, we did not compare HRQoL of covid patients with those of healthy adults. At the time the study was conducted, our entire hospital was transformed to a covid hospital, the outpatients visits were completely suspended thereby making any comparison impossible to be done.

## Conclusions

Overweight and hypertension are frequent coexisting conditions in young to middles age adults with COVID-19. Respiratory distress and hypokalaemia at the infection onset such as neurological and/or mental disorders are commonly associated with progressive disease. Regardless of hospitalization, either physical or mental status are deeply affected in the early stage of post-discharge.

## Data Availability

The datasets used and/or analyzed during the current study are available from the corresponding author on request.

## Abbreviations

COVID-19: New coronavirus disease 2019
HRQoL: Health related quality of life
ED: Emergency department
SF-36: Short form health survey
NMDs: Neurological and/or mental disorders
BMI: Body mass index
SARS-Cov-2: Severe acute respiratory syndrome coronavirus 2
RT-PCR: Reverse transcriptase-polymarase chain reaction
MERS: Middle east respiratory syndrome
SARS-Cov1: Severe acute respiratory syndrome coronavirus 1
IQR: Interquartile range
WHO: World Health Organization
